# Estimates of Effective Population Size in Commercial and Hatchery Strains of Coho Salmon (*Oncorhynchus kisutch* (*Walbaum*, 1792))

**DOI:** 10.3390/ani12050647

**Published:** 2022-03-03

**Authors:** Victor Martinez, Phillip J. Dettleff, Nicolás Galarce, Cristian Bravo, Jessica Dorner, Robert N. Iwamoto, Kerry Naish

**Affiliations:** 1 FAVET-INBIOGEN, Universidad de Chile, Avda. Santa Rosa 11735, La Pintana, Santiago 8820808, Chile; cl.bravo.v@gmail.com (C.B.); jdorner@ug.uchile.cl (J.D.); 2 Facultad de Medicina Veterinaria y Agronomía, Universidad de las Américas, Av. Walker Martínez 1360, La Florida, Santiago 8242125, Chile; satryl@veterinaria.uchile.cl; 3 Escuela de Medicina Veterinaria, Facultad de Ciencias de la Vida, Universidad Andrés Bello, Santiago 8370146, Chile; ngalarce@ug.uchile.cl; 4 Northwest Fisheries Science Center, Seattle, WA 98195, USA; iwamoto4944@msn.com; 5 School of Aquatic and Fishery Sciences, University of Washington, Box 355020, Seattle, WA 98195, USA; knaish@uw.edu

**Keywords:** coho salmon, effective population size, genetic variation

## Abstract

**Simple Summary:**

Several populations of Coho salmon have been maintained in aquaculture, but the extent of the genetic diversity in these strains is unknown. This paper describes the genetic status of several aquaculture strains of Coho salmon from North America, Chile, and Japan and a wild-type hatchery strain from the Pacific Northwest of North America. The Chilean strains in particular have been subject to changes in population sizes attributable to their establishment, reductions due to disease outbreaks, and maintenance of small population sizes in culture. An assumption-free method for estimating the changes in genetic diversity showed that many aquaculture strains had reduced variability. These results highlight the importance of monitoring the genetic diversity of aquaculture species from the start of breeding programs to secure their future genetic variation, particularly in challenging environments such as those expected from climate change.

**Abstract:**

Understanding the genetic status of aquaculture strains is essential for developing management guidelines aimed at sustaining the rates of genetic gain for economically important traits, as well as securing populations that will be robust to climate change. Coho salmon was the first salmonid introduced to Chile for commercial purposes and now comprises an essential component of the country’s aquaculture industry. Several events, such as admixture, genetic bottlenecks, and rapid domestication, appear to be determinants in shaping the genome of commercial strains representing this species. To determine the impact of such events on the genetic diversity of these strains, we sought to estimate the effective population size (Ne) of several of these strains using genome-wide approaches. We compared these estimates to commercial strains from North America and Japan, as well as a hatchery strain used for supportive breeding of wild populations. The estimates of Ne were based on a method robust to assumptions about changes in population history, and ranged from low (Ne = 34) to relatively high (Ne = 80) in the Chilean strains. These estimates were higher than those obtained from the commercial North American strain but lower than those observed in the hatchery population and the Japanese strain (with Ne over 150). Our results suggest that some populations require measures to control the rates of inbreeding, possibly by using genomic information and incorporating new genetic material to ensure the long-term sustainability of these populations.

## 1. Introduction

Coho salmon (*Oncorhynchus kisutch*) is one of the key salmonids contributing to aquaculture production in Chile. The species was first introduced to Chile from the west coast of North America in 1930, and since then, additional introductions have taken place from North America and Asia [1]. Farming began in the late 1980s, and Chile is now the leading producer of large Coho salmon worldwide [2].

Many existing aquaculture strains of Coho salmon are descended from few sources and share similar breeding goals [1]. However, many of these strains have been introduced into novel environments, often with associated founder events. Therefore, comparisons between these populations serve as a critical resource for examining modes of evolution and characterizing the extent of changes in genetic diversity since foundation. For example, the detection of signatures of selection across the genome can help identify genes underlying quantitative traits that may have played a role in unique or parallel processes [3]. Examining genetic diversity across populations can also provide important information on the capacity of individual strains to respond to ongoing environmental change. Many contemporary domesticated strains of Coho salmon originated from a few source populations in the Pacific Northwest in the USA and Canada. They have subsequently been introduced into different environments in southern Chile from the USA and Japan, with ongoing external introductions early in their history [1,2,4]. Most strains have been in culture in established breeding programs for about ten generations and have experienced intense natural and artificial selection. Therefore, they serve as a valuable model for comparative analyses.

Rapid changes in effective population size (Ne) are expected in commercial salmonid aquaculture due to inadequate management of broodstock. The establishment of breeding populations might not have been accompanied by methods to prevent genetic bottlenecks and high rates of inbreeding. Such events might have led to sudden changes in effective population sizes [5]. Salmonids can also be subjected to very high selection intensities, due to the species’ high fecundity rates. Genetic improvement of Chilean strains has been achieved through the implementation of an animal model, based on Best Linear Unbiased Prediction (BLUP). However, these practices might have increased the rates of inbreeding because populations that rely on fewer breeding families can lead to skewed genetic contributions. The consequences of co-selection of relatives on effective population size has not been well studied across Coho salmon populations [6]. The impact of natural selection on genetic diversity is also likely significant in Chilean salmonid aquaculture, attributable in part to *Piscirickettsia salmonis*, a bacterium inducing significant mortalities since culture began [7]. The disease produced by this bacterium has been called “coho salmon syndrome”, since it was first isolated from this species [8,9].

It is essential to understand the status and genetic sustainability of commercial populations of Coho salmon. This situation is critical since Coho salmon is not native to Chile, but genetic rescue using introductions from outside the country has not been permitted to prevent large-scale disease outbreaks resulted in significant losses to the Atlantic salmon industry. Local feral strains might serve a role in enhancing strains, but it is not known to what extent such a step is necessary. Thus, understanding the current genetic status of these populations can help develop plans for increasing the genetic sustainability of the commercial aquaculture of this species. In this paper, we present estimates of recent and historical effective population sizes obtained from different strains of Chilean Coho salmon and compare these with commercial strains in North America and Japan. We also include a source population that has not been subject to deliberate domestication selection. The use of both contemporary and historical measures across study populations provides an understanding of the dynamics of effective population size throughout the management history of each strain.

## 2. Materials and Methods

### 2.1. Commercial and Hatchery Strains

Samples from 5 Chilean strains that belong to individual companies (sample size between 20 and 45 individuals each, see Table 1) were surveyed with the previously developed Coho salmon single nucleotide polymorphisms (SNP) array [10]. These populations have been primarily subjected to selection for growth rate (using mass or BLUP selection) and maintained as closed populations for several generations. These strains were originally founded from different sources. Some were commercial, founded from populations in the Pacific Northwest in USA and Canada, including the Domsea strain in Washington [11] and Kitimat strain [12] in Canada [2]. Other sources originate from naturalized populations in Lago Llanquihue, which in turn are thought to have been made up of stocks from Oregon Aquafoods (Puget Sound and Oregon-Alsea, USA), Fish Pro and Kitimat (Canada), and Aquamar (Chile) [1]. In many cases, the exact origin of the Chilean strains is uncertain [2]. Short-term concerns of inbreeding were used initially to avoid mating between close relatives, but this is not well documented. The generation interval in captivity is around two years in Chile. Here, individuals were sampled within generations.

Data from a Japanese breeding population were obtained from ddRAD sequences of Coho salmon from the Inland Fisheries Experimental Station, Miyagi Prefecture Fisheries Technology Center (Miyagi, Japan) [13]. This population was established in 1978 from the Lower Kalama hatchery (Washington, WA, USA) and maintained for several generations without deliberate selection. Family identification began in 2000, where artificial selection was applied for less than two generations. Here, samples were obtained from the 2009 broodstock populations. The generation interval of this population was four years, although some cohorts used precocious males at three years of age [13]. Publicly available ddRAD sequences from 112 randomly selected individuals (sequences were not individually described) were downloaded from NCBI (Submission: DRA005759; BioProject: PRJDB5730).

The North American reference, the Domsea strain, was initially derived from wild Skykomish Coho salmon returning to the Wallace hatchery in 1971, followed by three generations of mass selection by the Washington Department of Fish and Wildlife (WDFW) in 1971 and 1972 [14]. The founding population was based on 40 families and 600 individuals, accompanied by high survival and growth rate in captivity [15]. A breeding design based on avoiding of matings between close relatives and use of a circular mating design was applied throughout the strain’s history to limit the rates of inbreeding [11]. The generation interval is two years [16], and at the time of sampling, the Domsea strain had been in a selection program for 20 generations or 40 years. Individuals were sampled across two separate lineages (even and odd year), and the results are presented within generations. The source population for the Domsea strain, Wallace hatchery, is a locally derived population [4,14] that returns to the Wallace River, a tributary of the Skykomish River, and is used to enhance the population size of wild fish within the system.

A summary of the data describing the different populations is presented in Table S1.

### 2.2. Genotypic Data

After sampling of fin tissue, DNA was extracted using the DNeasy Blood & Tissue Kits (Qiagen, Valencia, CA) from fin clips obtained from broodstock from the Chilean and North American strains. The Affymetrix SNP-chip used for genotyping contained 220,001 SNPs, as described previously [10]. Thermo Fisher carried out genotyping in Santa Clara, (CA).

The ddRAD sequences from the Japanese Coho salmon strain [13] were mapped to the Coho salmon genome version 2 (GCA_002021735.2, University of Victoria, Victoria, Canada) using the software BWA [17]. SAM files were sorted with samtools [18] and PCR duplicates were removed using SAMBAMBA software [19]. SNPs were identified using FreeBayes with default settings. The initial set of SNPs identified was filtered using vcftools [20] to a smaller group using the following criteria (derived from the FreeBayes output): (1) a Phred-scaled SNP quality score with significance greater than 30; (2) maximum allele frequency of 0.49; (3) maximum percentage of missing values of 0.20; and (4) the maximum number of alleles as 2. Missing values were imputed using Beagle version 5.2 [21].

### 2.3. Estimation of Linkage Disequilibrium and Effective Population Size (Ne)

Estimates of the extent of linkage disequilibrium (LD), r^2^ [22], were obtained using PLINK [23], using the default parameters. Linkage Disequilibrium decay for each chromosome arm was assessed using *e* estimated at different inter-marker physical distances, from 0.1 to 2 Mb and using a minimum allele frequency (MAF) of 0.05. Bins of physical distance (every 10 kilobases) were constructed using the “content” method as implemented in the software ONeR [24].

Estimates of Ne were obtained using the software GONE [25]. The approach minimizes the sum of the squared differences between the observed values of *d*^2^ (Ohta and Kimura 1969; Rogers 2014). These values are obtained as the average of r^2^ [22] between pairs of SNPs, weighted by their variance in allele frequencies across several bins given different ranges of chromosomal distances in Morgans [26]. The method uses a genetic algorithm to infer the temporal series of Ne in the population that minimizes the sum of the squared differences between the observed values of *d*^2^ of the bins, and those predicted considering different demographic histories. Notably, the temporal estimates of Ne are not modelled with the underlying assumption that there is a linear relationship between Ne and generation time, the main assumptions associated with other estimation methods [27]. The analyses assumed that phase was unknown. The chromosomal distances between SNPs were assumed to follow a linear relationship between physical distance and cM (1 cM1 Mb), corrected using the *kosambi* mapping function over short distances (c = 0.05). The position of the SNPs was obtained by aligning the harbouring sequence of each SNP with the Coho salmon genome version 2 (CF_002021735.2) using BWA [17].

For comparison purposes, estimates of Ne were also obtained from an approach that assumes a constant linear relationship between Ne and the number of generations. The method used the derivations of [27,28] and has been implemented in the software SNeP by [29]. Default parameters were used in all the populations analyzed.

## 3. Results

### 3.1. Linkage Disequilibrium (LD)

#### Marker Data

After final filtering, a set of 189,501 segregating SNPs with a minor allele frequency (MAF) over 0.01 and below 0.49 across all screened populations was selected from the Coho salmon genotyping array. The median minor allele frequency was 0.24. A total of 147,349 SNPs were mapped to the 30 autosomes and used for the final analysis. The median marker spacing was 4.4 kilobases. Missing values were imputed using Beagle version 5.2 [21].

In comparison, the median of the SNP marker distance for the ddRAD data on the Japanese population was 8.0 kilobases. The average number of markers per chromosome for the ddRAD dataset was 1727, giving a total of 51,794 across the Coho salmon genome. A description of sample size, number of polymorphic SNPs genotyped, and minimum allele frequencies are presented in Table 1.

**Figure 1:**
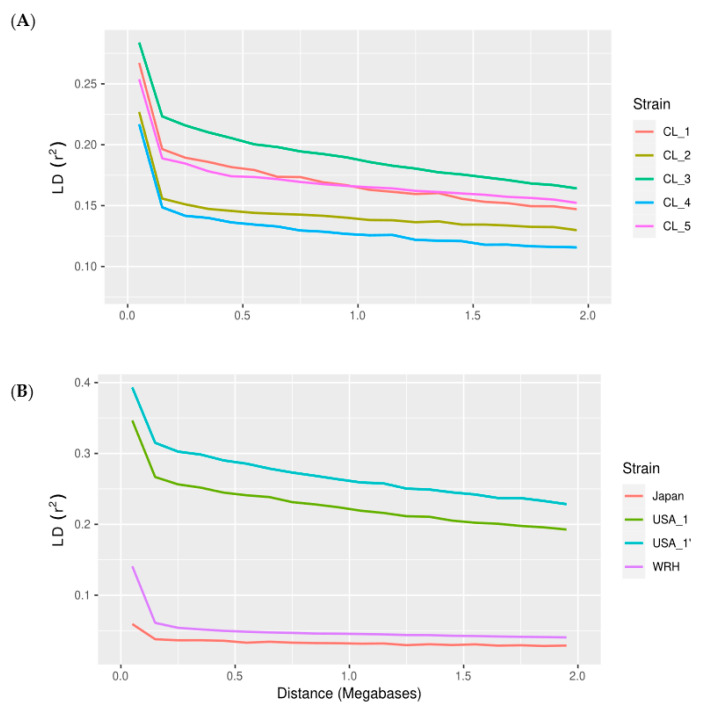
Decay of the average linkage disequilibrium (LD) genome-wide, measured as r^2^ against physical distance (Mb): (**A**) Chilean strains, and (**B**) North American strains and Japanese strains.

The relationship between LD decay and physical distance (Mb) is presented within each population over distances up to 2 Mb (Figure 1). In general, the average LD (r^2^) decay was most pronounced between SNPs separated within 0.15 Mb (Figure 1). This result was observed across all populations. For example, the r^2^ decayed at half the initial values from 0.2 to 2 Mb in the Chilean strains. However, on average, the r^2^ values of the North American commercial Domsea lines was higher than the values observed in the Chilean populations. In contrast, the r^2^ values were small and decreased rapidly in the Wallace River hatchery and Japanese populations. The maximum average value of r^2^ was almost half those in the commercial populations, and the lowest average r^2^ was obtained from the Japanese population.

### 3.2. Patterns of Effective Population Size, Ne, across Generations

**Figure 2:**
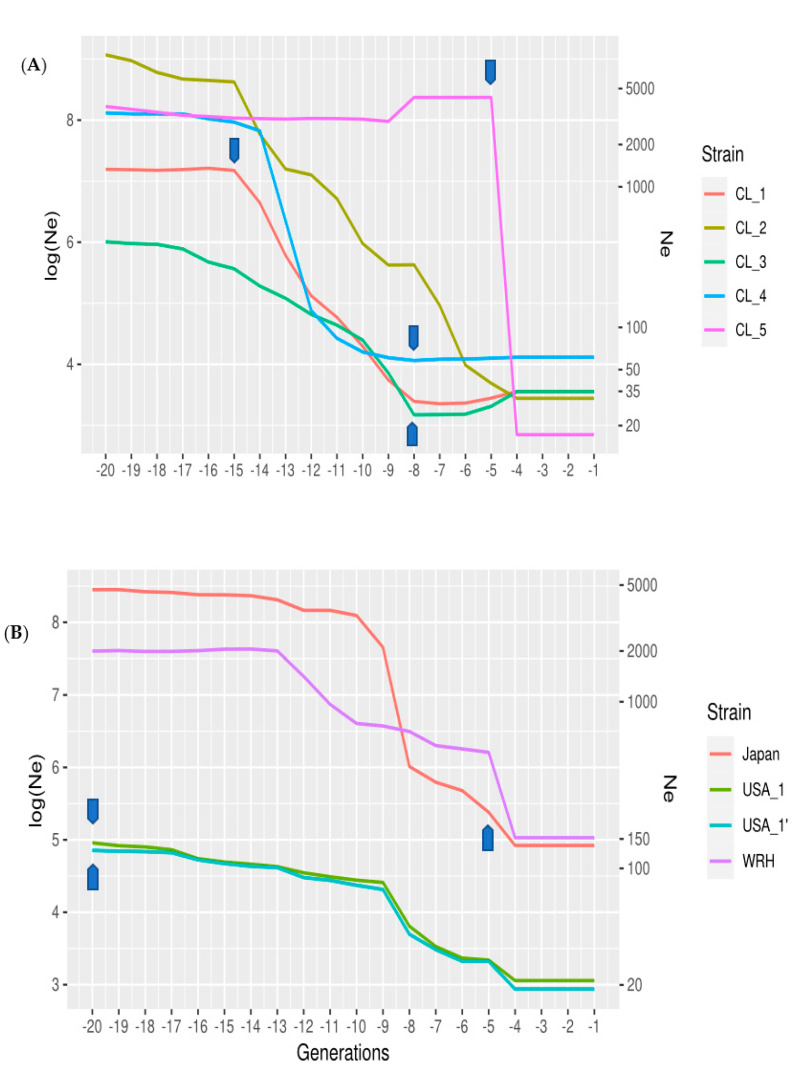
Estimates of the effective population size (Ne) on a logarithmic scale (log), second Y axis actual Ne) within the last twenty generations, reflecting the time over which different strains were established. Each strain was analyzed using the methods of [25]: (**A**) Chilean strains, and (**B**) North American strains and Japanese strains. Arrows (Blue) denote the most likely generation when the breeding program was started (Table S1).

The Chilean commercial strains showed a non-linear pattern of change in Ne across time (Figure 2), reflecting the complexity of the breeding history of Coho salmon in Chile. Most of the Chilean populations had Ne values between 20 and 60 in the most recent generations (Figure 2). Three strains, CL_1, 2, and 4, shared a bottleneck 14 to 15 generations prior to sampling, after which two of the strains (1 and 2) experienced a gradual decline in Ne, to stabilize in more recent generations. CL_4 continued to decline sharply to 12 generations, after which the Ne stabilized to give the highest contemporary values. In contrast, strain CL_5 retained a high effective size until 4 generations prior to sampling, when it experienced a dramatic decline in Ne (from almost 5000 to 17; Figure 2). Finally, the Ne values for strain CL_3 declined gradually since founding until 8 generations ago, after which the effective population size increased.

Changes in Ne in the commercial Domsea lines of North America were smaller across time. However, the relationship between Ne and the generations was not linear. There was a tendency towards decreased Ne values from 20 generations until nine generations in the past, after which there was a greater rate of decline from nine to four generations ago. This change is very similar between both lines (even and odd; Figure 2; USA_1 and USA_1’). In the most recent generations, the Ne in the North American commercial strain was the smallest of all the different strains analyzed (Ne = 25). The contemporary effective population size in the wild-type Wallace River hatchery strain was the largest in all the strains analyzed, with values of Ne of about 165, although there was a significant decrease from 12 to 4 generations ago. Within the Japanese strain, the greatest drop in effective population size occurred seven and three generations prior to sampling.

**Figure 3:**
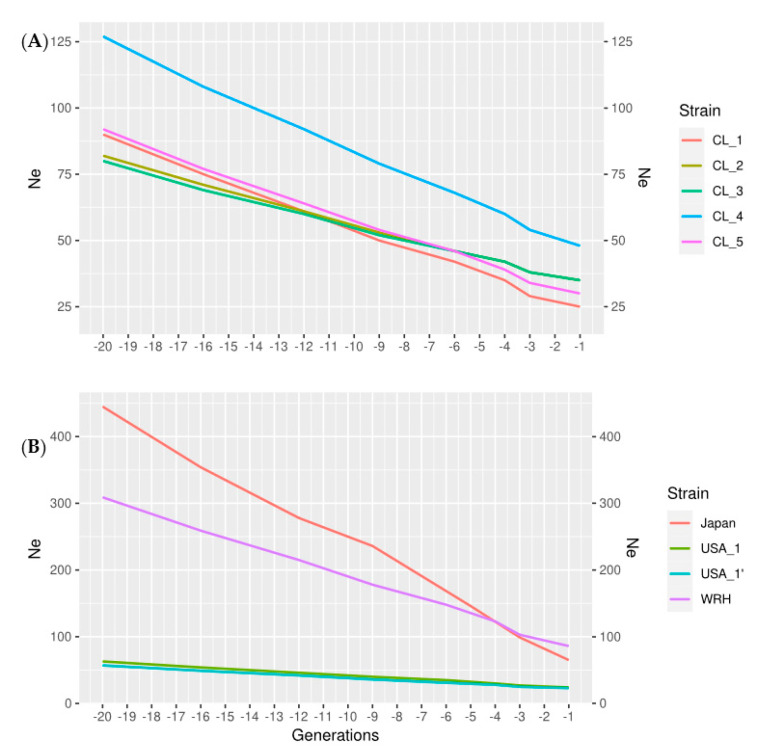
Estimates of Ne within the last twenty generations, obtained using the methods of [27]: (**A**) Chilean strains, and (**B**) North American strains and Japanese strains.

The method of Corbin [29] provided lower estimates of Ne (Figure 3); this method assumes a constant or linear relationship between Ne and time. Since this method could not accurately model the complex patterns of population history in aquaculture, some downwards bias can be expected (see Discussion, [26]). The Japanese commercial strain had amongst the higher estimates of effective population size (Ne= 65 based on the method of Corbin [27] and Ne = 137 based on the technique of Santiago [25]) in the recent past. The strain shows a sudden decrease in Ne, about three generations ago, which is expected based on the population history of the strain (about five generations ago, a mass selection program was started based on growth rate).

## 4. Discussion

The main goal of breeding programs is to increase the profitability and sustainability of the breeding population. In aquaculture, increased fecundity can often result in a large census population at the expense of a relatively low effective population size. The asymmetry of genetic contributions within breeding programs can also be influenced by the biology of the species, such as spawning behavior [30], or the application of intense selection without efforts to constrain the long-term rates of inbreeding [15]. Recent studies have shown that relatively low effective population sizes in aquaculture are the norm in marine species and salmonids, but overall, the estimates are scarce [26,31]. The significance of measuring Ne for aquaculture populations is that it provides a measure of the likelihood of genetic gain. Here, we found that the contemporary effective population size is relatively low in most of the strains we studied, and the Chilean strains in particular revealed the genetic consequences of a range of management scenarios. Some strains shared historical population bottlenecks (about 15 generations prior to sampling), and exhibited a subsequent slow decline in Ne. However, these values have stabilized in more recent generations, possibly a result of improved management. The effective population sizes in one strain remained stable historically, but experienced a dramatic decline more recently. However, the majority of the Chilean strains had higher contemporary effective population sizes and lower linkage disequilibrium compared to a strain that has been maintained longer in culture—the Domsea line. The Wallace River hatchery population and the Japanese strains had larger estimates of Ne. Most of the commercial strains had Ne values below 50, recognized as the minimum value for reducing the effects of inbreeding and when genetic drift can significantly counteract the effects of natural selection [32]. Importantly, these values provide guidelines for comparative assessment of these populations’ sustainability and genetic risk, and whether it would be necessary to implement methods to further constrain the rates of inbreeding [30].

The contemporary and historical estimates of effective population size across strains provides insight into their different management histories. The Domsea lines from the USA have been deliberately managed to limit the rates of inbreeding by using circular mating designs and avoidance of mating between relatives [13]. However, the population has remained closed since founding. The rate of decline in Ne was largely constant over time, reflecting the inevitable consequences of maintaining a relatively small population in captivity. This result is in contrast to those observed in the Chilean populations. Here, companies established formal Coho salmon breeding programs relatively recently, the majority no more than ten generations ago. In general, breeding programs were founded using many families in the nucleus population (from 100 to 200 full-sib families), which may explain the relatively large effective population size obtained in earlier generations in several strains. While some companies mixed eggs from different hatcheries, others based their selective breeding programs on closed broodstock populations that they had already established. Mass selection was initially used in many breeding programs, and pedigree records were not maintained. Therefore, the constraint of long-term inbreeding rates was not initially considered. As a result, large decreases in the effective population size were observed over time, particularly in some of the strains analyzed (see Figure 2). Further, *Piscirickettsia salmonis* produced increased mortalities, which were particularly impactful when their culture began in Chile [7,33]. Such mortalities may explain the shared bottlenecks in the early generations, especially if these strains shared common origins (some before they were formally founded). Interestingly, the founding of two new strains subsequent to these events, in generation eight, appears to have resulted in stabilization or improvement of Ne (see Figure 2); these populations have been managed to avoid mating between relatives (Table S1). However, the exact history of these populations is uncertain, and there are a range of other explanations for changes in genetic diversity. For example, the use of a few families for spawning due to management or environmental reasons (such as volcano eruptions in the south of Chile, [34]) could not be ruled out. The Japanese strain had higher contemporary values of Ne, possibly reflecting the fact that they were maintained for several generations as a randomly mating population without deliberate selection, prior to formal management [13].

The results of this study also provide information relevant to conservation management. The Wallace River hatchery population has been maintained over time to support returning wild salmon and, as such, has incorporated locally derived wild fish in the hatchery [14]. This population has not been artificially selected, but it is possible that the hatchery environment has resulted in genetic changes through processes such as genetic drift or selection. For example, hatchery broodstock may rely on a few individuals, resulting in a low effective population size. The release of large numbers of hatchery fish can in certain circumstances reduce the Ne of the wild population (the Ryman–Laikre effect) [35]. In addition, the reproductive success of hatchery fish in the wild can be lower than naturally born fish [36,37], possibly as a result of domestication selection. Taken together, these factors may explain some of the changes in *Ne* seen here [4]. To our knowledge, the estimates presented here are the first to model recent changes of Ne, using an assumption-free robust method in a hatchery population, such as that found in the Wallace hatchery. Therefore, monitoring changes in effective population size can help assess sudden changes or increases of this important parameter on the basis of genomic data, without having to rely on pedigrees.

The LD values estimated between markers at short distances provide insight into the possible effects of selection. Here, the values obtained for the commercial populations (≤0.1 Mb) is very similar to r^2^ values reported for other aquaculture species, such as rainbow trout lines raised in France [31]. In this case, LD declined rapidly within the first 100 kb (mean r^2^ decreased from 0.34 at 10 kb to 0.23 at 100 kb) [31]. At longer distances between markers (e.g., about 1 Mb apart), the observed LD (0.13–0.18) was similar to that observed in the Chilean lines. This result is likely to be explained by the selection intensity applied at the start of breeding programs in these populations. Overall these values are lower than the ones observed in terrestrial species [38].

The small effective population sizes observed here are relevant to understanding future scenarios in aquaculture. For example, the ability to adapt to climate change can impact the future viability of aquaculture and natural populations [39,40,41]. The likelihood for adaptation of salmonids can be explained by long- and short-term exposure to increased water temperature. Population bottlenecks due to sudden changes in water temperature may be cumulative, therefore limiting their long-term evolutionary potential. These situations have been observed in natural populations where changes in heat tolerance were small but significant [39]. Population recovery could be too slow in species with long generation intervals that have undergone drastic reductions in population size, such as some of the populations analyzed here. Therefore, measures to maintain genetic variation in cultured populations should be considered a priority and addressed when possible through genomic selection [10]. This situation is particularly critical in the North American and Chilean strains with relatively low Ne, which may in turn restrict the evolutionary potential of these strains. No eggs can be imported from outside of the country due to stringent disease management protocols.

Concerns have been raised when applying Ne estimation methods to populations created by admixture, or when generating synthetic populations. The method of estimating Ne used here [25] has been shown to be robust to population admixture in predicting recent population history, demonstrated using simulations [26]. Further, Ne estimates in an admixed pig population (created by mating, synthetic lines) were comparable to pedigree estimates using the method of [25]. Some of the Chilean strains were developed using eggs purchased from different Canadian and American hatchery populations. Therefore, early broodstock management in some lines was not exclusively performed on a specific line or sourced from a single egg supplier [2]. After this initial period, the consolidation of specific breeding programs began, and the strains were maintained as closed populations for several generations after these initial admixture events. Data from this study suggests that some Chilean strains were admixed. This effect may explain the large initial Ne values, followed by sudden and very significant decreases (particularly in one Chilean strain); however, further investigation on this topic is required to assess the extent of admixture between the different lines analyzed.

An important issue is how accurate the estimates of Ne are when considering different methods. Estimates from GONE are proportional to the sample size, the square root of the number of pairs of SNPs included in the analysis, and the inverse of the effective population size [25]. The sample size in this study varied between 20 and 112 individuals (Table 1). However, the amount of information used to estimate Ne in this study appears to be sufficient to obtain relatively accurate estimates_._ Here, we used more than 147 thousand SNPs within relatively small recombination rates (c = 0.05), providing sufficient power to obtain relatively accurate estimates. This large number of marker pairs at different distances was combined with relatively large sample sizes, gave enough power to estimate Ne. Estimates with as few as 32 haplotypes (n = 16) appear to be sufficient to recover population history, particularly recent events, as demonstrated using simulations and real data [25]. The lowest number of markers is the one obtained from the ddRAD dataset, but the sample size of this population was more than double that of the Chilean strains, and is in the same order of magnitude of estimates obtained in other studies as well as those obtained using simulations [25].

It is necessary to point out that any estimation method of Ne has several assumptions. In many instances, these assumptions may not be fully met in practice. For example, the estimates obtained from [27,29] assume a constant or linear relationship between Ne and generation number. Estimates of Ne based on this method were in all cases much smaller than those predicted based on the more assumption-free estimate derived from the method of [25]. Simulations showed that the method of [27] gave consistently downward biased estimates when the populations did not follow a constant or linear decrease in Ne, which is the case for the aquaculture strains. In this study, most Chilean strains had Ne values near Ne = 25 using these methods; much smaller estimates were obtained for the North American commercial strains (Ne = 18). Both the Japanese and the North American hatchery strain had recent values of about 65, which are nearly three times smaller than those obtained using [25]. Moreover, these estimates from recent generations relied upon longer physical genomic distances and were subjected to large standard errors [27]. Therefore, these estimates should be interpreted with caution, given the history of these populations.

## 5. Conclusions

Commercial Coho salmon strains have undergone a series of events leading to contemporary values of Ne below 50 in some populations, which is the minimum value recommended for short-term sustainability of selection programs and avoiding genetic risks associated with inbreeding [6,32]. The estimates of Ne in the Chilean populations reveal a significant decrease in the past, probably due to processes associated with establishment and domestication, exposure to diseases, and high selection intensities in breeding programs. Our results suggest that some populations require measures to control the rates of inbreeding, possibly by using genomic information and by incorporating genetic material from other populations. The management of effective population size is important in cases where variation in climate change can have consequences for the long-term sustainability of these populations. However, in the short term, loss of genetic diversity is of significant concern. Intervention is essential for minimizing the loss of alleles through genetic drift, improving the ability of populations to respond to selection and increase disease resistance, and for managing inbreeding depression.

## Figures and Tables

**Table 1:** Description of the SNP data obtained from the different populations analyzed. Sample size, number of SNPs genotyped, and the median minimum allele frequency (MAF). SNPs were derived from the Coho salmon microarray, except for the Japanese population, where SNPs were obtained from double digested Restriction-Site Associated DNA (ddRAD) loci.

Population	Abbreviation	Number of samples	Number of SNPs Genotyped	Median MAF
Chile Strain 1	CL_1	45	146,945	0.19
Chile Strain 2	CL_2	20	135,663	0.23
Chile Strain 3	CL_3	44	138,893	0.31
Chile Strain 4	CL_4	40	135,868	0.21
Chile Strain 5	CL_5	36	135,743	0.28
Domsea Even	USA_1	30	135,624	0.17
Domesea Odd	USA_1′	25	136,297	0.18
Wallace River	WRH	46	146,288	0.24
Japan	JAPAN	112	51,794	0.10

## Data Availability

The data is available upon request to the corresponding author.

## Supplementary Materials

The following are available online at https://www.mdpi.com/article/10.3390/ani12050647/s1, Table S1: Biodata of the populations used in the analysis.
