# Supplementary material for: Estimates of Effective Population Size in Commercial and Hatchery Strains of Coho Salmon (Oncorhynchus kisutch (Walbaum, 1792))
Source: Animals (Basel). 2022 Mar 3;12(5):647. doi: 10.3390/ani12050647 (PMC8909777; doi:10.3390/ani12050647)
Supplement: Supplementary file 1 [file animals-12-00647-s001.zip › animals-1567120-supplementary.pdf]

Table S1. Biodata of the populations used in the analysis.

Acronym	Generations of the breeding program	Traits selected	Inbreeding management
**CL_1**	15	Harvest weight	-
**CL_2**	-	-	-
**CL_3**	8	Growth	Avoidance of sib matings
**CL_4**	8	Growth	Avoidance of sib matings
**CL_5**	5	Not known	Incorporating eggs from other companies.
**USA_1 (even line)**	21	The selection was primarily for growth and survival	Circular matings and avoidance matings between close relatives
**USA_1' (odd line)**	20	The selection was primarily for growth and survival	Circular matings and avoidance matings between close relatives
**WRH**	-	Natural selection at the hatchery environment	-
**Japan**	2	Harvest weight [13]	No information
